# Cost-effectiveness of hemodialysis and peritoneal dialysis: A national cohort study with 14 years follow-up and matched for comorbidities and propensity score

**DOI:** 10.1038/srep30266

**Published:** 2016-07-27

**Authors:** Yu-Tzu Chang, Jing-Shiang Hwang, Shih-Yuan Hung, Min-Sung Tsai, Jia-Ling Wu, Junne-Ming Sung, Jung-Der Wang

**Affiliations:** 1 Institute of Clinical Medicine, College of Medicine, National Cheng Kung University, Tainan, Taiwan; 2 Division of Nephrology, Department of Internal Medicine, National Cheng Kung University Hospital, College of Medicine, National Cheng Kung University, Tainan, Taiwan; 3 Institute of Statistical Science, Academia Sinica, Taipei, Taiwan; 4 Division of Nephrology, Department of Internal Medicine, E-DA Hospital, and School of Medicine for International Students, I-Shou University, Kaohsiung; 5 Division of Nephrology, Department of Internal Medicine, Kuo General Hospital, Tainan, Taiwan; 6 Department of Public Health, College of Medicine, National Cheng Kung University, Tainan, Taiwan; 7 Department of Environmental and Occupational Health, National Cheng Kung University Hospital, College of Medicine, National Cheng Kung University, Tainan, Taiwan

## Abstract

Although treatment for the dialysis population is resource intensive, a cost-effectiveness analysis comparing hemodialysis (HD) and peritoneal dialysis (PD) by matched pairs is still lacking. After matching for clinical characteristics and propensity scores, we identified 4,285 pairs of incident HD and PD patients from a Taiwanese national cohort during 1998–2010. Survival and healthcare expenditure were calculated by data of 14-year follow-up and subsequently extrapolated to lifetime estimates under the assumption of constant excess hazard. We performed a cross-sectional EQ–5D survey on 179 matched pairs of prevalent HD and PD patients of varying dialysis vintages from 12 dialysis units. The product of survival probability and the mean utility value at each time point (dialysis vintage) were summed up throughout lifetime to obtain the quality-adjusted life expectancy (QALE). The results revealed the estimated life expectancy between HD and PD were nearly equal (19.11 versus 19.08 years). The QALE’s were also similar, whereas average lifetime healthcare costs were higher in HD than PD (237,795 versus 204,442 USD) and the cost-effectiveness ratios for PD and HD were 13,681 and 16,643 USD per quality-adjusted life year, respectively. In conclusion, PD is more cost-effective than HD, of which the major determinants were the costs for the dialysis modality and its associated complications.

The increased number of patients with chronic kidney disease (CKD) worldwide is a growing threat to public health and healthcare systems^1^. The progressive course of CKD will ultimately result in end-stage renal disease (ESRD), which necessitates dialysis or transplantation to maintain patients’ lives. Patients with ESRD usually have various comorbidities, which not only consume substantial healthcare resources for management, but also further deteriorates their quality of life (QOL) and survival rates. Around 1.2–6.0% of the annual health care budget of developed countries, including Taiwan, is spent for the clinical management of ESRD patients, who only represent 0.01–0.30% of their national populations^2^,^3^,^4^. Because of rapidly aging populations and decreased mortality rates over the past few decades^3^, we anticipate an increase in the prevalence rates and financial burden for patients with ESRD. In the United States (US), it is estimated that the total healthcare expenditure spent on ESRD will be 53.6 billion US dollars in 2020. It is a 2.5-fold of increase when compared to the costs in 2005^5^. The total healthcare expenditure for ESRD patients is known to be mainly driven by the costs for the dialysis procedure itself^2^, with a similar phenomenon also being observed in Taiwan^6^. Since there are at present few effective strategies to control the occurrence of ESRD, this will increase the financial difficulties faced by healthcare insurance systems. A cost-effective approach to the choice of dialysis modality is thus necessary, not only to minimize the financial burden of the healthcare insurance systems, but also to improve QOL and survival.

Renal transplantation is the most cost-effective renal replacement therapy for ESRD^2^,^7^,^8^. However, the shortage of organ sources limits its application, and most ESRD patients thus end up receiving either hemodialysis (HD) or peritoneal dialysis (PD) throughout their lifespan. HD and PD are well-established and mature treatment modalities for ESRD patients, with the former being performed by trained professionals three times a week to remove uremic toxins via dialyzers, and the latter being performed by patients or their caregivers every day to eliminate uremic toxins via the peritoneal membrane. The choice of HD or PD as the initial dialysis modality is related to multiple factors, including government or reimbursement policy, multiple comorbidities, personal lifestyle, accessibility of HD or PD, incomplete presentation of dialysis choices, and nephrology experiences^9^,^10^,^11^. In general, patients with the following conditions are not favored for PD for fear of more infectious complications or technical difficulties: those with severe neurological or psychological illnesses and without the help of caregivers, extensive abdominal adhesion, or poor personal hygiene. While variations are found in high-, middle- or low-income nations^2^, the cost of PD usually seems to be lower than that of HD, as is the case in Taiwan^12^. However, the use of HD is more prevalent than PD in many countries.

Although numerous studies have evaluated the costs^2^,^8^,^13^, survival function^14^,^15^,^16^,^17^,^18^,^19^,^20^,^21^,^22^,^23^,^24^,^25^,^26^,^27^ and QOL^28^,^29^,^30^,^31^,^32^,^33^,^34^,^35^ between HD and PD, the majority usually evaluated these outcomes and costs separately, and the results from these works have not been entirely consistent because of numerous confounding factors^36^, especially the presence of concomitant comorbidities. Till now, comparative cost-effectiveness studies of HD and PD among patients with similar clinical conditions are lacking. Although a large scale randomized controlled trial would be the best solution to deal with this problem, this option is too difficult to undertake^29^. An alternative solution is the use of a matching process to control potential confounders. In this study, a matched-pair study was conducted to compare the cost-effectiveness between HD and PD by estimating quality-adjusted life expectancy (QALE) and cost-per-quality-adjusted life year (QALY). As shifting modalities during follow-up might also confound the results, we have restricted the selection of subjects to those receiving only HD or PD. The evidence provided in this study can help policy makers and clinicians with regard to prioritizing dialysis modalities.

## Methods

### Establishment of the national cohort of the dialysis population

**Figure 1:**
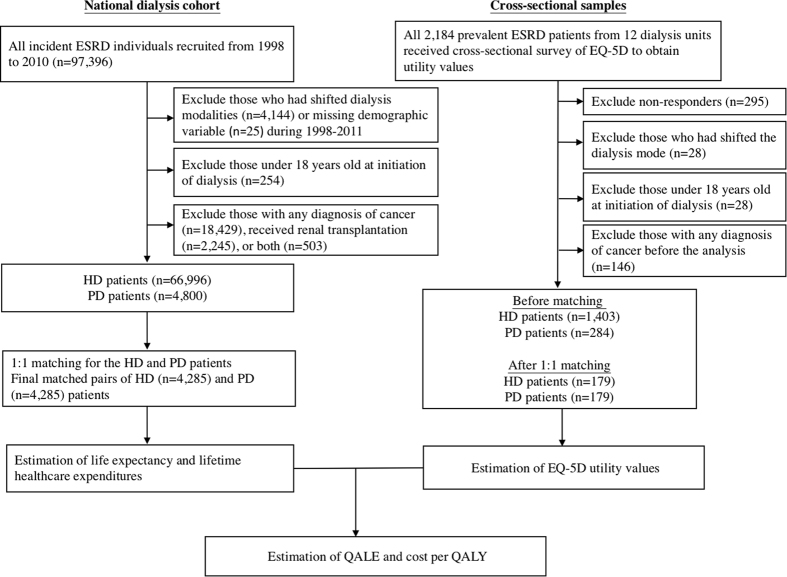
Flow diagram of establishing the study cohorts and cross-sectional samples followed by matching on clinical characteristics and propensity scores for patients under hemodialysis (HD) and peritoneal dialysis (PD) to estimate quality-adjusted life expectancy (QALE) and lifetime costs. Abbreviation: ESRD: end-stage renal disease; QALE: quality-adjusted life expectancy; QALY: quality-adjusted life year.

This study was approved by the ethics review board of National Cheng Kung University Hospital (A-ER-101-089) before commencement, and the methods were carried out in accordance with the approved current guidelines. The Taiwan National Health Insurance (NHI) program was initiated in March 1995, and by 2001 it provided medical services for more than 99% of the 23 million residents in Taiwan^37^. Nearly every kind of medical service can be reimbursed by the NHI, including all payments for outpatient and inpatient services, medication prescriptions and intervention procedures. Each beneficiary is required to pay a certain amount of copayment toward the utilization of the medical service. The rate of copayments ranges from less than 10% for usual outpatient visits to the highest rate of 30% for prolonged hospitalization. However, patients with specific diseases on the list of catastrophic illness, including ESRD on maintenance dialysis therapy, can be waived from copayment. Therefore, each patient with any catastrophic illness must be validated by at least two specialists to avoid abuse of the NHI program. Moreover, the waiving of copayments for ESRD under maintenance HD and PD makes our estimation of total cost comprehensive and accurate. This study was conducted using the reimbursement database of the NHI. We performed a nationwide collection of incident ESRD patients (International Classification of Diseases, Ninth Revision, Clinical Modification codes [ICD-9 codes]: 585) with certifications of catastrophic illness related to dialysis therapy (HD or PD). Patients older than 18 years and receiving dialysis for more than three consecutive months from January 1, 1998, to December 31, 2010, were enrolled in the dialysis cohort (Fig. 1). The dates of the enrollment of dialysis patients were the first date of receiving dialysis for three consecutive months. HD and PD therapies were identified according to their procedure codes. The survival of each dialysis patient was verified by cross-linkage with the database of the National Mortality Registry. Because patients with malignancy (ICD-9 codes: 140-208, 230-234) are usually associated with high mortalities, poor QOL and high subsequent costs of management for the malignancy and associated complications. In addition, there is a great variation in life expectancies (LEs), QOL and healthcare expenditures among malignancies of different organ-systems and stages. Therefore, we excluded all patients with malignancy to avoid potential confounding of estimations by cancer (Fig. 1). In addition, patients who had ever changed the dialysis mode for more than three months were also excluded because of the difficulty to quantify the effects of shifting dialysis modality at different dialysis vintages on survival, costs and QOL. Since many patients receiving renal transplantation were first treated with dialysis while waiting for an appropriate donor, they were treated as censored on the date of transplantation. However, we excluded all patients with previously performed transplantation before dialysis to avoid any potential bias in this study due to prior transplantation. Major comorbidities noted before initiation of dialysis were identified with the corresponding ICD-9 codes (listed in Supplementary Table 1), as follows: If the patient had any of these comorbidities in the hospitalization discharge codes once, or at least twice in ambulatory care with 30 days apart, within one year. Such procedures for analyzing the NHI database could improve the accuracy of measurement of comorbidities and have contributed to many high quality studies^38^,^39^,^40^,^41^. The end of the follow-up period was December 31, 2011.

### Collection of information of EQ-5D by cross-sectional sampling in prevalent dialysis patients

From February 2012 to May 2013, we conducted a cross-sectional survey of prevalent dialysis patients from 12 dialysis centers to collect the information of QOL. Informed consent was obtained from all study participants. We applied the EQ-5D to assess health-related QOL, which has five domains and can be converted into utility values for estimation of QALY^42^. Each patient was assessed by a researcher assistant who was experienced in conducting the EQ-5D survey. When necessary and applicable, information of the EQ-5D was obtained from the family members or primary caregivers of the patients as proxies. The utility value of EQ-5D ranged from 0 to 1, where 0 indicates death and 1 indicates perfect health^43^. The concomitant diseases of patients that were noted while interviewing were recorded as comorbidities. As before, patients with malignancy, dialysis mode switching or aged under 18 years old when visiting were excluded from the analysis.

### Matching based on individual characteristics and propensity scores

Since the choice of dialysis modality of the physician and/or patients is based on minimizing mortality and likelihood of developing complications, as well as improving QOL, or, the overall outcomes, we first matched the two groups on major comorbidities that would result in premature mortality, and this was then followed by a matching of propensity scores to minimize potential residual confounding. The whole process thus takes the preference of physician and/or patients into consideration. A 1:1 matching of incident HD and PD patients from the national dialysis cohort was performed for age (±2 years), sex, index year of initiation of dialysis, urbanization status, and major comorbidities, including diabetes mellitus, acute myocardial infarction, congestive heart failure, stroke, chronic liver disease, and the propensity scores up to ±0.05. The propensity score for PD prescription was estimated with a logistic regression model^44^,^45^, which included hypertension, coronary artery disease, cardiac dysrhythmias, peripheral vascular disease, hyperlipidemia, and rheumatological disease as independent variables. These baseline characteristics were chosen for matching because of their association with LEs and healthcare expenditures, which were the parameters of cost-effectiveness^21^,^23^,^24^,^46^ (Fig. 1). To control the confounding of QOL, we conducted 1:1 matching on the cross-sectional samples interviewed for QOL measurement, i.e., age (±5 years), sex, duration of dialysis (±3 months), diabetes mellitus, cardiovascular disease, stroke, chronic liver disease and propensity score for PD (±0.05), of which the independent variables included hypertension, hyperlipidemia, and rheumatological disease (Fig. 1). Similarly, these characteristics were chosen because of the association with QOL in dialysis patients^47^,^48^,^49^,^50^. In addition, a wider range of age-matching criteria was applied in the cross-sectional samples because of the relatively homogenous distribution of utility values within each five-year strata (Supplementary Table 2).

### Estimation of LE of dialysis population

After the 1:1 matching from the national dialysis cohort, a total of 8,570 incident HD and PD patients were identified. We first estimated the survival function of these subjects using the Kaplan-Meier method during the follow-up period of 14 years. The lifetime survival function was then extrapolated up to 720 months by a semiparametric method under the assumption of constant excess hazard^51^,^52^,^53^ from the end of the 14^th^ year. The bootstrap method of utilizing 100 repeated samples was performed to obtain the standard error of means. The estimation was achieved using the iSQoL software^54^. The accuracy and application of this method have been validated in various studies in cohorts of different illnesses^55^,^56^,^57^,^58^, including dialysis populations^59^,^60^,^61^.

### Estimation of QALE of the matched pairs from the national dialysis cohort

**Figure 2:**
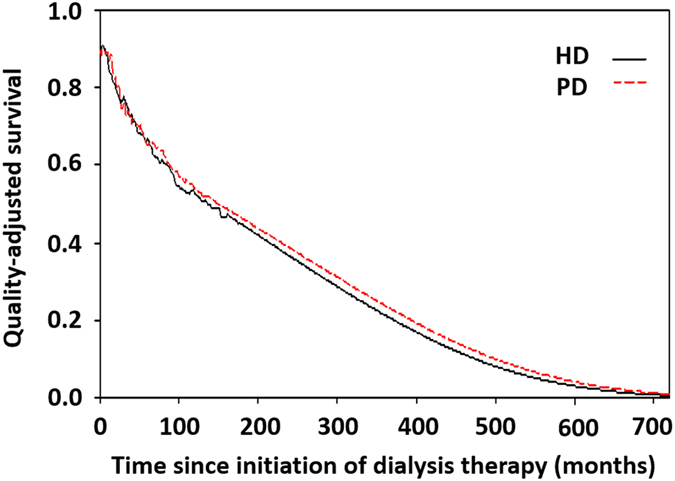
Estimation of quality-adjusted life expectancy (QALE) for patients under hemodialysis (HD) and peritoneal dialysis (PD). Lifetime survival curves of HD and PD were depicted in the black and red lines, respectively. The QALE was estimated by summarizing the total area under the survival curve.

We multiplied the survival probability at each time point (dialysis vintage) with the mean utility value of QOL and summed up throughout lifetime estimates to obtain the QALE (Figs 1 and 2). Kernel smoothing using a nearest neighbor approach was applied to average the nearest 10% of the utility values of the interviewed dialysis patients. The sample size should be at least over 50 for a random sample^62^. After this, the lifetime survival function was adjusted by the corresponding mean QOL function to obtain a quality-adjusted survival curve, of which the sum of the area under this curve was the QALE of dialysis patients.

### Estimation of lifetime healthcare expenditures, cost per QALY and incremental cost-effectiveness ratio (ICER) for HD and PD patients

We retrieved the total monthly medical costs reimbursed by the NHI, which were available during the period of 1998–2010 and included all healthcare expenditures for the inpatient and outpatient services, medications, examinations, and intervention procedures (e.g., dialysis), for every enrolled incident dialysis subject (n = 8,570) throughout this time period. That is, all medical costs after the initiation of dialysis therapy (including those for treating comorbidities, complications, and so on) were included, with the exception of the costs of transportation and hiring special caregivers. Summarizing any cost of medical service after the initiation of dialysis therapy can help us to clarify which dialysis modality induces higher subsequent costs, including the dialysis modalities themselves and their associated complications. These monetary values were first adjusted to those for 2010 according to the Consumer Price Index, and the average monthly cost was calculated for HD or PD for each month after beginning dialysis. The extrapolated NHI expenditures after 2010 were annually discounted at 3%, as recommended by the World Health Organization’s project entitled Choosing Interventions that are Cost–Effective (WHO-CHOICE)^63^. The total average monthly expenditures were then multiplied by the monthly survival probabilities for HD or PD and summed up throughout lifetime to estimate the lifetime healthcare expenditures. The equation for ICER (PD to HD) was calculated as follows: [Lifetime cost for (PD - HD)]/[QALE of (PD - HD)].

### Statistical analysis

Continuous variables were summarized as means ± standard deviations. Comparisons of continuous variables were assessed by using the Student’s t test and/or the Kruskal-Wallis test. The categorical variables were presented as the number of cases (and percentages), and the comparison of differences or trends between various groups was carried out using the Chi-square test, Fisher’s exact test or Mental-Haenszel Chi-square test. All statistical analyses were performed with SAS version 9.4 (SAS Institute, Cary, NC, USA). A two-sided *p* value < 0.05 was considered statistically significant.

## Results

### Baseline characteristics of the patients from the national dialysis cohort and the cross-sectional samples

We identified 71,796 incident cases of ESRD during 1998–2010 after exclusion. After matching with individual characteristics and propensity scores, we recruited 4,285 matched-pairs of incident HD and PD patients from the national dialysis cohort (Fig. 1). Among 2,184 prevalent patients non-selectively invited from 12 dialysis centers, 295 patients refused the EQ-5D survey. After excluding patients aged under 18 (n = 28), having dialysis mode switching or renal transplantation (n = 28) and having malignancy (n = 146), 1,403 HD and 284 PD patients were entered into the final analysis. Table 1 summarizes the differences in baseline characteristics among the national dialysis cohort, responders and non-responders of the cross-sectional samples. The responders from the cross-sectional samples were more likely to be younger and with fewer comorbidities than those of the national dialysis cohort in either HD or PD groups. Compared with the non-responders, the responders of HD patients were more likely to have lower proportions of education levels at “did not finish school” or “get married” (66.6% versus 77.1%, P < 0.001), having a lower “duration on dialysis” (74.3 versus 89.5 months, P < 0.001), and to show higher prevalences of hypertension (61.5% versus 52.1%, P = 0.003) and hyperlipidemia (6.2% versus 2.1%, P = 0.005). In the PD patient group, the distributions of baseline characteristics between responders and non-responders were similar.

### Baseline characteristics of the patients from the national dialysis cohort and the cross-sectional samples after matching for clinical characteristics and propensity scores

Table 2 showed there were no statistically significant differences between the matched pairs of incident HD and PD patients selected from the national dialysis cohort in age, sex, dialysis duration, urbanization and most of the comorbidities. However, PD patients had a lower prevalence of peripheral vascular diseases (9.4% versus 12.7%, standardized differences [di]:10.54), but showed slightly higher prevalences of hypertension (89.5% versus 85.8%, di:11.26) and hyperlipidemia (53.7% versus 46.7%, di:14.03). Among the matched pairs of prevalent dialysis individuals from the cross-sectional samples, there were no statistically significant differences in most of the clinical characteristics, except in dialysis duration, age, education level and employment status.

### Distribution of EQ-5D scores for each domain, utility values and EQ-5D visual analogue scales in prevalent ESRD patients on HD and PD therapy

In the EQ-5D survey among the total cross-sectional samples of prevalent dialysis patients, most PD patients reported no problems in mobility (86.6%), self-care (90.9%), usual activities (83.5%), pain/discomfort (68.0%) or anxiety/depression (75.0%) (Table 3). In contrast, only 71.3–74.3% HD patients reported no problems in self-care and anxiety/depression and 54.4–61.4% in mobility, usual activity and pain/discomfort. The mean EQ-5D values calculated by an equation established by Taiwan (TW)^64^ were also higher in patients with PD than with HD (0.90 versus 0.81, respectively). To facilitate an international comparison, a sensitivity analysis by replacing the utility values estimated by the United Kingdom (UK)-based equation^43^ and the scores of the visual analogue scale (0–100) showed the same trend.

After matching, HD patients had slightly higher proportions reporting no problems in all domains than PD patients, except in the domain of anxiety/depression. When converting the utility values by the TW-based equation, the mean utilities were nearly equal for both HD and PD patients (0.88 versus 0.89, respectively). Applying the utility values estimated by the UK-based equation and the scores of the visual analogue scale also showed no differences between the matched HD and PD patients. In addition, we added employment status as one more criterion for matching and re-ran the analysis, which resulted in 144 pairs for HD and PD with utility values based on the TW function of 0.88 and 0.88, respectively (Supplementary Tables 3 and 4), thus indicating robust results for QOL measurements.

### LE, QALE, lifetime healthcare expenditures, and cost-effectiveness analysis between patients on HD and PD therapy

**Figure 3:**
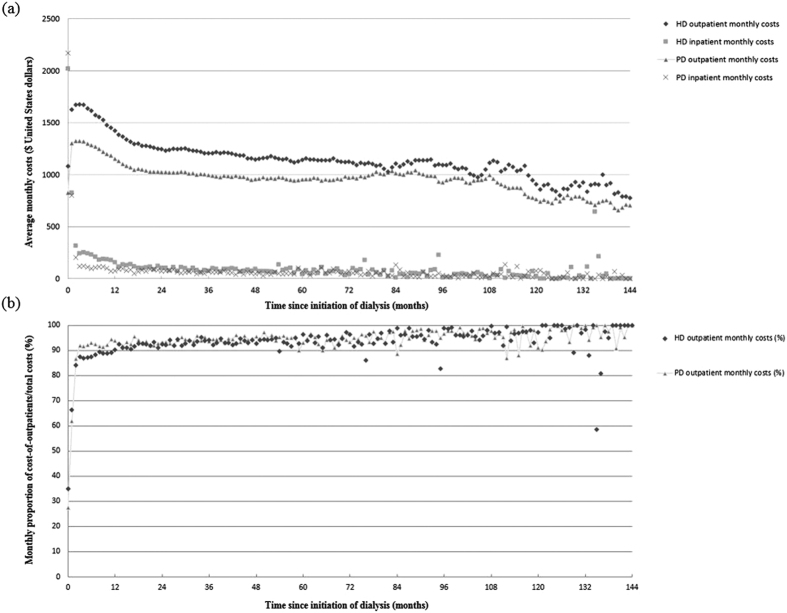
(**a**) Average monthly costs for inpatient and outpatient healthcare expenditures and (**b**) monthly proportions of outpatient costs within total healthcare expenditures of patients selected from 1:1 matched hemodialysis (HD) and peritoneal dialysis (PD) during the follow-up period.

The estimated LE of HD and PD patients were nearly equal (19.11 versus 19.08 years, Table 4). After adjusting for the utility values from matched samples, we found that the QALE of HD and PD patients were similar (16.42 versus 17.41 QALY, respectively, P = 0.072) (Table 4 and Fig. 2). The average lifetime cost paid by the NHI for HD was higher than that of PD (237,795 versus 204,442 USD). During the follow-up period, we found that the monthly average cost of outpatient services (including dialysis) accounted for about 90% of the total cost, and PD was almost always less costly with regard to monthly total healthcare expenditures, as shown in Fig. 3. This indicates a higher dialysis cost and/or higher frequency of outpatient visits for HD, and the complications requiring hospitalization in PD were not more severe than those in HD throughout the observation period. After adjusting for a 3% annual discount rate for both denominator and numerator, the cost-effectiveness ratios for PD and HD therapy were 13,681 and 16,643 USD per QALY, indicating savings of nearly 3,000 USD per QALY for PD compared with HD. The cost per QALY of both HD and PD represented 0.74- and 0.90-fold the value of gross domestic product per capita of Taiwan in 2010, which indicates dialysis therapy is cost-effective, in line with the suggestion of the WHO-CHOICE project^63^. The estimated ICER of PD to HD was −50,858, which further supports the dominance of PD in the cost-effectiveness analysis.

### Sensitivity Analysis

The sensitivity analysis, performed using 2:1 matching, showed largely the same results as those observed in the 1:1 matching. The distributions of baseline characteristics between HD and PD after matching were almost the same (Supplementary Table 5). Although there was no significant difference for survival between HD and PD during the follow-up period (Supplementary Figure 1), the estimated LE was longer for HD than for PD (19.03 vs. 18.01 years, respectively) (Supplementary Table 6). After adjustment for survival by QOL, there was no difference in QALE between patients with HD and those with PD (16.33 vs. 16.43 QALY, respectively). The dominance of PD over HD increased from about 3,000 USD per QALY to 5,000 USD per QALY.

## Discussion

This study adopted a matching design for a nationwide incident dialysis population followed for 14 years and comprehensively estimated lifetime survival function, costs, and dynamic changes of QOL, which might solve most of the potential limitations of previous studies. For patients without switching modes, our results show that PD is more cost-effective than HD if the dialysis cost of HD is higher than that of PD. This is strongly corroborated by the following arguments: First, since we closely matched PD and HD patients on sex, age, major comorbidities and propensity scores, this study controlled as many potential confounders as possible that might account for the differences of survival and QOL functions and preference for dialysis modalities, which may have improved the internal validity. Second, the application of utility values throughout duration-to-date with a kernel smoother could account for the dynamic change in QOL over time. Third, since all dialysis patients registered in the catastrophic illnesses in Taiwan can be waived from co-payment, and the total follow-up period is more than a decade, the total healthcare expenditures estimated in this study would be very comprehensive and information bias would be minimized for both PD and HD. Since the resulting survival and QOL functions for PD and HD were almost the same after matching (Table 4), the major differences in the cost-effectiveness ratios mainly came from the different costs of different modalities and the associated complications. As the dialysis cost under PD is less expensive than HD in Taiwan, PD is dominant over HD with regard to cost-effectiveness, as is also seen in Fig. 3 (and Supplementary Figure 2).

Numerous studies have been conducted to compare the survival outcomes between patients receiving HD and PD^14^,^15^,^16^,^17^,^18^,^19^,^20^,^21^,^22^,^23^,^24^,^25^,^26^,^27^. Although the messages from these studies were mixed and sometimes confusing, the majority indicated that HD and PD conferred similar survival rates, despite some differences that existed within the distinct subgroups of the patients^65^. In our study, the comparison of the survival function of the 1:1 matched pairs from the national dialysis cohort during the 14-year follow-up period revealed no statistical difference, as evaluated by the Kaplan-Meier method and log-rank test (Supplementary Figure 3). The sensitivity analysis of applying 1:2 matching showed consistent results (Supplementary Figure 1). Therefore, our study corroborates the current consensus opinion that PD was associated with a similar risk of mortality compared to HD.

The underutilization of PD therapy has been noted for decades. One of the possible reasons for this might be related to the debates with regard to long-term survival and secular change in QOL after initiation of HD or PD. In addition, the costs for PD are generally lower than HD in most countries^2^. However, the possible selection bias of HD and PD patients may account for this difference. In contrast to previous studies, we have taken advantage of the inter-linkages among different national databases and applied close matching as a major strategy to control the above biases. Given that we have shown the equivalence of survival and QOL after the close matching of HD with PD and the less costly nature of PD (Table 4, Fig. 3 and Supplementary Figure 2), we conclude that PD is dominant in the cost-effectiveness analysis. As 71–76% of dialysis patients are suitable for PD therapy^66^,^67^,^68^, our evidence can be used for policy makers to reconstruct the imbursement policy for the promotion of PD in clinical practice for those patients without contraindication.

The heavy financial burden of dialysis therapy on health care systems has led to numerous economic evaluations of different dialysis modalities^7^,^13^,^69^,^70^, and a Markov model is commonly used in evaluating the cost-effectiveness of these approaches. One of the basic assumptions of Markov model is the use of fixed sets of transition probabilities, health care costs, and QOL, which are usually derived from the data of a short follow-up period. In reality, these parameters are time-dependent^71^,^72^,^73^,^74^, which would increase the inaccuracy of the resulting estimations, and a sensitivity analysis is always needed to cover the uncertainty. As there have been no randomized control trials with a large sample for HD and PD^29^, the parameters applied in the Markov model are generally subject to the selection bias of HD and PD in the general dialysis population. In this study, we directly measured the monthly survival rates and average costs of medical services after initiation of dialysis for more than a decade (Fig. 3 and Supplementary Figure 3). In addition, the kernel-smoothing average estimation method takes the dynamic change in QOL over time into account^62^ (Supplementary Figure 4). As such, our estimates might more accurately catch the real patient-centered conditions and be closer to the actual situation.

This study showed a generally higher mean EQ-5D utility value of both prevalent HD and PD (0.83 and 0.90, respectively) than those of the previously published reports (HD group: 0.44−0.71; PD group: 0.53–0.72)^35^,^75^. Such a difference might be related to the younger age and fewer comorbidities in our cross-sectional sample (Table 1), and the fact that there is no copayment for dialysis under the NHI system of Taiwan, which may reduce the financial pressure and psychological stress of dialysis patients. In fact, one previous study from Taiwan revealed that the scores for the item “health and social care availability” and the environment domain of WHOQOL-BREF were even better in HD patients than in those of healthy referents^50^, which indicates the satisfaction of dialysis patients with the NHI system. Nonetheless, a Taiwanese nationwide EQ-5D survey was performed in 2009, which showed a significantly higher average utility value of the general population of 0.931 after matching sex and age with our dialysis sample, or, a mean utility of 0.824 (Supplementary Table 7). Furthermore, we conducted this survey during 2011–2, and so the advances in dialysis technology and medical care over time and different cultural systems^76^ may also be responsible for the differences. Since both national PD and HD cohorts were generally older and showed higher proportions of comorbidities than those of the cross-sectional samples (Tables 1 and 2), the utility values in this study may be overestimated, and one must be cautious in making generalizations from our study results to the whole dialysis population.

Because of low copayment, convenience and nearly full coverage of all medical services, most citizens in Taiwan are strongly adherent to the NHI program, and it provides the government and our team an opportunity to comprehensively estimate the total healthcare expenditures based on the reimbursement database. The 2012 total healthcare expenditure per capita in Taiwan was 1,350 USD^77^, which is only 15.3% of the value in the United States, 28.6% of Germany, 28.2% of Japan and 37.6% of the United Kingdom^78^. As we have also taken the 3% discount rate into consideration, the final estimated lifetime healthcare expenditure and cost-per-QALY in our study appears much lower than those seen in these high income countries. Since the average cost for each PD service is usually lower than that of HD in many high income countries, we would expect that the resulting difference in lifetime healthcare expenditures and costs-per-QALY between PD and HD would also be higher in these countries.

There are several limitations to our analyses, as follows. First, we excluded patients who received transplantation or shifting dialysis and performed individual matching to control differences in risk-factor distributions of survival function and QOL across different dialysis modalities. Though this process may improve the internal validity of the comparison between PD and HD, it might make it less applicable to generalize to the whole dialysis population; however, the detailed data in Tables 1 and 2 provide a basis for valid comparison and possible adjustment. Since the above procedures only excluded 6.6% and 1.3% of the national cohort with ESRD and patients eligible for assessing QOL, respectively, they would at most lead to a minimal bias and would not change the conclusion of PD dominance. Second, compared with the 1:1 matched national cohorts (n = 4285 in Table 2), the matched cross sectional sample (n = 179) were slightly younger in age and had lower prevalence rates of comorbidities (including diabetes, cardiovascular diseases, and liver cirrhosis), which could possibly lead to the overestimation of the QOL values for both PD and HD. Since these values were comparable for both dialyses, they would not likely change the conclusion of PD dominance. In contrast, our sensitivity analysis of adding employment status as one more criterion for matching resulted in a small sample size (n = 144), which also showed similar results (or, 0.88 vs. 0.88 for HD vs. PD, as in Supplementary Tables 3 and 4). Therefore, we expect that any change in utility values resulting from the selection criteria that improved the validity of the comparison would be unlikely to affect the conclusion of PD dominance. Further, we assumed that the values of QOL were constant and the same as those at the end of the follow-up period in order to estimate the value of QOL measured beyond the longest follow-up period in our cross-sectional samples. This could lead to the overestimation of QOL within this period because of the gradual decline of QOL during the aging process. However, the estimation bias would be minimized with the concomitant decrease in the survival rate after 14 years of dialysis. Third, the estimated expenditures did not include costs of family care and opportunity costs of the patients. In light of the flexible dialysate exchange program, patients on PD are more likely to keep their employment status and spend less time and money on transportation to dialysis centers. Therefore, the difference in costs between HD and PD would be larger if we took the opportunity costs into account. Fourth, although we have tried our best to match major comorbidities and propensity scores in this study, we still cannot completely rule out residual confounding resulted from choices and decisions involved in the selection process of dialysis modalities. For example, we do not have data on body mass index or hemoglobin level available in the two databases for our study, although both factors are related to survival function, healthcare expenditure and QOL. Besides, the lack of information with regard to decision making for dialysis modalities, such as the incentives offered by the reimbursement policy or personal lifestyle factors, might also leave some residual confounding and/or selection bias.

Despite its limitations, this study shows that the survival and QOL functions were almost the same for HD and PD after matching. The superiority of PD in this study resulted from its generally lower overall cost compared with HD. Since the total healthcare expenditures, including costs of inpatient and outpatient services, for dialysis modalities are mainly driven by the costs of the dialysis procedure itself^2^, our conclusions can probably be generalized to countries with higher expenditures for HD than PD per dialysis session. The potential saving of opportunity costs for PD further increases its superiority over HD, especially among those patients with regular employment. However, future studies are warranted to explore the cost-effectiveness of shifting dialysis modalities.

## Additional Information

**How to cite this article**: Chang, Y.-T. *et al*. Cost-effectiveness of hemodialysis and peritoneal dialysis: A national cohort study with 14 years follow-up and matched for comorbidities and propensity score. *Sci. Rep.* **6**, 30266; doi: 10.1038/srep30266 (2016).

## Supplementary Material

Supplementary Information

## Figures and Tables

**Table 1:** Comparison of demographic and clinical characteristics of dialysis patients among the national cohort, respondents and non-respondents of the cross-sectional sample stratified by modality (HD: hemodialysis, PD: peritoneal dialysis).

		HD			P value*	PD			P value*
		National cohort	Cross-sectional samples			National cohort	Cross-sectional samples		
Characteristics			Respondents	Non-respondents			Respondents	Non-respondents	
**Number of patients**	66996		1403	288		4800	284	7	
**Age, No (%)**									
Mean (SD)		62.1 (13.7)	57.1 (13.6)	55.5 (15.4)	0.103	53.1 (15.0)	46.7 (13.2)	46.7 (17.1)	0.991
18–34 years		2170 (3.2%)	87 (6.2)	28 (9.7)	0.213	572 (11.9%)	59 (20.8)	3 (42.9)	0.071
35-49 years		10301 (15.4%)	338 (24.1)	72 (25.0)		1358 (28.3%)	112 (39.4)	2 (28.6)	
50–64 years		23133 (34.5%)	562 (40.1)	102 (35.4)		1765 (36.8%)	90 (31.7)	0 (0.0)	
65–79 years		25359 (37.9%)	367 (26.2)	75 (26.0)		905 (18.8%)	19 (6.7)	2 (28.6)	
≥80 years		6033 (9.0%)	49 (3.5)	11 (3.8)		200 (4.2%)	4 (1.4)	0 (0.0)	
**Sex (No of male, %)**		33260 (49.6%)	700 (49.9)	130 (45.1)	0.142	2156 (44.9%)	145 (51.1)	2 (28.6)	0.279
**Level of education completed, No (%)**									
Did not finish school		—	276 (19.7)	81 (28.1)	0.005	—	5 (1.8)	0 (0.0)	0.825
Elementary school		—	568 (40.5)	88 (30.6)		—	48 (16.9)	1 (14.3)	
Junior high school		—	197 (14.0)	49 (17.0)		—	41 (14.4)	2 (28.6)	
Senior high school		—	245 (17. 5)	50 (17.4)		—	113 (39.8)	3 (42.9)	
University and above		—	117 (8.3)	20 (6.9)		—	77 (27.1)	1 (14.3)	
**Marriage status (No of married, %)**		—	934 (66.6)	222 (77.1)	<0.001	—	191 (67.3)	4 (57.1)	0.688
**Duration of dialysis (months)**		55.2 (39.3)	74.3 (61.0)	89.5 (71.0)	<0.001	46.7 (29.8)	42.6 (37.1)	28.3 (29.4)	0.393
**Employment, No (%)**		—	406 (28.9)	131 (45.5)	<0.001	—	158 (55.6)	4 (57.1)	0.937
**Urbanization**					—				—
0 (Rural)		21919 (32.7%)	—	—		1260 (26.3%)	—	—	
1 (Satellite)		19072 (28.5%)	—	—		1397 (29.1%)	—	—	
2 (Metropolitan)		26005 (38.8%)	—	—		2143 (44.6%)	—	—	
**Comorbidities (%)**									
Diabetes mellitus		62.3	39.1	36.8	0.461	41.8	31.3	57.1	0.215
Hypertension		90.1	61.5	52.1	0.003	89.3	83.8	66.7	0.260
Cardiovascular disease		70.3	23.1	21.9	0.654	47.0	17.6	0.0	0.608
Stroke		25.3	6.2	8.7	0.123	13.2	2.5	0.0	1.000
Hyperlipidemia		49.8	6.2	2.1	0.005	53.7	33.8	71.4	0.052
Rheumatological disease		5.6	1.3	1.0	0.736	7.5	3.2	0.0	1.000
Chronic liver disease		28.2	19.2	16.7	0.321	24.3	14.8	28.6	0.286

Abbreviation: SD: standard deviation; HD: hemodialysis; PD: peritoneal dialysis.

^*^P values were calculated in the comparison between respondents and non-respondents under the same modality.

**Table 2:** Comparison of demographic and clinical characteristics between dialysis patients after matching for individual characteristics, major comorbidities, and propensity score stratified by hemodialysis (HD) and peritoneal dialysis (PD).

Characteristics	Matched samples from national cohort			Matched cross-sectional sample		
	HD	PD	di	HD	PD	di
**Number of patients**	4285	4285		179	179	
**Dialysis duration (month)**	46.5 (29.6)	46.9 (29.8)	1.35	49.2 (37.7)	42.2 (37.5)	18.62
**Age, Mean (SD)**	54.3 (14.0)	54.2 (14.1)	0.71	50.5 (12.1)	49.1 (12.6)	11.33
** No (%)** 18–34 years	374 (8.7)	376 (8.8)	0.35	19 (10.6)	22 (12.3)	5.34
** **35–49 years	1202 (28.0)	1209 (28.2)	0.44	68 (38.0)	76 (42.5)	9.19
** **50–64 years	1662 (38.8)	1667 (38.9)	0.21	70 (39.1)	62 (34.6)	9.34
** **65–79 years	877 (20.5)	863 (20.1)	0.99	21 (11.7)	16 (8.9)	9.22
** **≥80 years	170 (4.0)	170 (4.0)	0.00	1 (0.6)	3 (1.7)	10.33
**Sex (male, %)**	1946 (45.4)	1946 (45.4)	0.00	88 (49.2)	88 (49.2)	0.00
**No. of married (%)**	—	—	—	131 (73.2)	129 (72.1)	2.47
**Level of education, No (%)**			—			
Did not finish school	—	—		18 (10.1)	4 (2.2)	33.34
Elementary school	—	—		62 (34.6)	33 (18.4)	37.34
Junior high school	—	—		35 (19.6)	30 (16.8)	7.26
Senior high school	—	—		43 (24.0)	63 (35.2)	24.72
University and above	—	—		21 (11.7)	49 (27.4)	40.39
**Employed, No (%)**	—	—	—	78 (43.58)	106 (59.22)	31.68
**Urbanization status**						
0 (Rural)	1108 (25.9)	1108 (25.9)	0.00	—	—	—
1 (Satellite)	1243 (29.0)	1243 (29.0)	0.00	—	—	
2 (metropolitan)	1934 (45.1)	1934 (45.1)	0.00	—	—	
**Comorbidities, No (%)**						
Diabetes mellitus	1863 (43.5)	1863 (43.5)	0.00	58 (32.4)	58 (32.4)	0.00
Hypertension	3675 (85.8)	3834 (89.5)	11.26	152 (84.9)	154 (86.0)	3.12
Cardiovascular disease	2124 (49.6)	1982 (46.3)	6.61	28 (15.6)	28 (15.6)	0.00
Congestive heart failure	941 (22.0)	941 (22.0)	0.00	—	—	—
Coronary artery disease	1255 (29.3)	1239 (28.9)	0.88	—	—	—
myocardial infarction	139 (3.2)	139 (3.2)	0.00	—	—	—
Cardiac dysrhythmias	605 (14.1)	567 (13.2)	2.62	—	—	—
Peripheral vascular disease	544 (12.7)	401 (9.4)	10.54	—	—	—
Stroke	497 (11.6)	497 (11.6)	0.00	3 (1.7)	3 (1.7)	0.00
Hyperlipidemia	2001 (46.7)	2301 (53.7)	14.03	28 (15.6)	28 (15.6)	0.00
Rheumatological disease	298 (7.0)	295 (6.9)	0.39	5 (2.8)	3 (1.7)	7.42
Chronic liver disease	967 (22.6)	967 (22.6)	0.00	24 (13.4)	24 (13.4)	0.00

Abbreviation: SD: standard deviation; di: standardized differences.

**Table 3:** Frequency distributions of various EQ-5D domain scores, utility values and visual analogue scales (VAS) for cross-sectional samples of dialysis patients after and before matching.

EQ-5D score	Matched samples			Total cross-sectional samples		p-value
	HD (n = 179)	PD (n = 179)	di*	HD (n = 1403)	PD (n = 284)	
Mobility dimension, %						<0.001†
No problems	152 (84.9)	137 (76.5)	21.41	862 (61.4)	246 (86.6)	
Some problems	25 (14.0)	40 (22.3)	21.66	480 (34.2)	34 (12.0)	
Confined to bed	2 (1.1)	2 (1.1)	0.00	61 (4.4)	4 (1.4)	
Self-care dimension, %						<0.001†
No problems	159 (88.8)	150 (83.8)	14.58	1043 (74.3)	258 (90.9)	
Some problems	13 (7.3)	22 (12.3)	16.88	260 (18.5)	17 (6.0)	
Unable to wash/dress	7 (3.9)	7 (3.9)	0.00	100 (7.1)	9 (3.2)	
Usual activities dimension, %						<0.001†
No problems	146 (81.6)	127 (71.0)	25.12	764 (54.4)	237 (83.5)	
Some problems	26 (14.5)	44 (24.6)	25.68	509 (36.3)	38 (13.4)	
Unable to perform	7 (3.9)	8 (4.5)	2.99	130 (9.3)	9 (3.2)	
Pain/discomfort dimension, %						<0.001†
None	120 (67.0)	114 (63.7)	6.94	774 (55.2)	193 (68.0)	
Moderate	54 (30.2)	61 (34.1)	8.36	584 (41.6)	84 (29.6)	
Extreme	5 (2.8)	4 (2.2)	3.84	45 (3.2)	7 (2.5)	
Anxiety/depression dimension, %						0.310†
None	135 (75.4)	142 (79.3)	9.33	1000 (71.3)	213 (75.0)	
Moderate	41 (22.9)	36 (20.1)	6.82	374 (26.7)	64 (22.5)	
Extreme	3 (1.7)	1 (0.6)	10.33	29 (2.1)	7 (2.5)	
Utility score by TW function						
Mean (SD)	0.88 (0.17)	0.89 (0.18)	5.71	0.81 (0.21)	0.90 (0.17)	<0.001‡
Median (IQR)	0.92 (0.18)	1.00 (0.10)		0.86 (0.27)	1.00 (0.10)	<0.001§
Range	0.26–1.00	0.10–1.00		0.00/1.00	0.00/1.00	
Utility score by UK function						
Mean (SD)	0.88 (0.15)	0.90 (0.16)	12.90	0.83 (0.19)	0.90 (0.16)	<0.001‡
Median (IQR)	0.90 (0.17)	1.00 (0.13)		0.87 (0.22)	1.00 (0.13)	<0.001§
Range	0.30/1.00	0.14/1.00		0.00/1.00	0.00/1.00	
EQ VAS, mean (SD)	67.3 (15.6)	69.9 (15.4)	16.77	65.4 (15.2)	69.4 (15.7)	<0.001‡
Median (IQR)	70.0 (20.0)	70.0 (20.0)		70.0 (20.0)	70.0 (20.0)	<0.001§
Range	0/100	10/100		0/100	10/100	

Abbreviation: SD: standard deviation; di: standardized difference; HD: hemodialysis; PD: peritoneal dialysis; TW: Taiwan; UK: United Kingdom.

^*^The comparison of difference between matched HD and PD patients by standardized differences.

^†^The comparison of difference between total cross-sectional HD and PD patients by the Mantel-Haenszel Chi-square test for trend.

^‡^The comparison of difference between total cross-sectional samples of HD and PD patients by the Student’s t test.

^§^The comparison of difference between total cross-sectional samples of HD and PD patients by the Kruskal-Wallis test.

**Table 4:** Comparison of cost-effectiveness for maintenance hemodialysis (HD) and peritoneal dialysis (PD): Lifetime survival functions and costs were estimated from 1:1 matched pairs of incident dialysis patients from the national dialysis cohorts (n = 4285 pairs) based on 14 years of follow-up, and utility values measured for prevalent dialysis patients from the cross-sectional samples after matching (n = 179 pairs).

	HD	PD	p-value
Life expectancy (in years) (SE)	19.11 (0.11)	19.08 (0.09)	0.853
Lifetime cost of NHI ± SE (US dollars)	237,795 ± 6,161*	204,442 ± 4,888*	<0.001
QALE (in QALY) (SE)	16.42 (0.47)	17.41 (0.24)	0.072
QALE (in QALY) (SE) (with 3% discount)	14.29(0.39)	14.94(0.2)	0.149
Cost per QALY ± SE (US dollars)	16,643 ± 659*	13,681 ± 354*	<0.001
ICER (PD-HD)	−50,858* (PD dominance)		

Abbreviations: NHI: National Health Insurance; SE: standard error of mean; QALE: quality-adjusted life expectancy; QALY: quality-adjusted life year; ICER: incremental cost-effectiveness ratio.

^*^The currency exchange rate is based on the value reported by the Central Bank of Taiwan on 2010/12/31: 1 United States Dollar = 30.368 New Taiwan Dollar. The gross domestic product per capita of Taiwan in 2010 is 18,573 US Dollar.
