# Supplementary material for: Cost-effectiveness of hemodialysis and peritoneal dialysis: A national cohort study with 14 years follow-up and matched for comorbidities and propensity score
Source: Sci Rep. 2016 Jul 27;6:30266. doi: 10.1038/srep30266 (PMC4962092; doi:10.1038/srep30266)

The title of the manuscript :

Cost-effectiveness of hemodialysis and peritoneal dialysis: A national cohort study with 14 years follow-up and matched for comorbidities and propensity score.

The author list:

Yu-Tzu Chang, MD, MSc,[1,2] Jing-Shiang Hwang, PhD,[3] Shih-Yuan Hung, MD,[4] Min-Sung Tsai, MD,[5] Jia-Ling Wu, MSc,[2] Junne-Ming Sung, MD,[2] and Jung-Der Wang, MD, ScD,[2,6,7]

Institutions:

From the [1]Institute of Clinical Medicine, College of Medicine, National Cheng Kung University, Tainan, Taiwan; [2] Division of Nephrology, Department of Internal Medicine, National Cheng Kung University Hospital, College of Medicine, National Cheng Kung University, Tainan, Taiwan; [3]Institute of Statistical Science, Academia Sinica, Taipei, Taiwan; [4]Division of Nephrology, Department of Internal Medicine, E-DA Hospital, and School of Medicine for International Students, I-Shou University, Kaohsiung; [5]Division of Nephrology, Department of Internal Medicine, Kuo General Hospital, Tainan, Taiwan; [6]Department of Public Health, College of Medicine, National Cheng Kung University, Tainan, Taiwan; [7]Department of Environmental and Occupational Health, National Cheng Kung University Hospital, College of Medicine, National Cheng Kung University, Tainan, Taiwan.

Supplementary Table 1. ICD-9-CM codes used to identify the associated comorbidities in the study.

Clinical diagnosis	The associated ICD-9-CM codes
Diabetes	250, 357.2, 362.0X, and 366.41.
Hypertension	401-402, 405, and A codes 260 and 269.
Cardiovascular disease:	
Congestive heart failure	398.91, 425, 428, 402.X1, 404.X1, and 404.X3.
Coronary artery disease	414.
Myocardial infarction	410.X, 412.
Cardiac dysrhythmias	426, 427.
Peripheral vascular disease	440–444, 447.1.
Stroke	
Ischemic stroke	433.xx, 434.xx, and 436.xx.
Hemorrhagic stroke	430.xx, 431.xx, and 432.xx.
Hyperlipidemia	272.0-272.4 and A code 189.
Malignancy	140-208, 230-234.
Rheumatologic disease	710.X, 714.X.
Liver disease	
Chronic liver disease	570, 571, 572.4
Hepatitis B virus infection	070.2, 070.3, and V02.61
Hepatitis C virus infection	070.7, 070.41, 070.44, 070.51, 070.54, and V02.62.

Supplementary Table 2. Mean utility values of the cross-sectional samples stratified by age and dialysis modalities.

	Hemodialysis		Peritoneal dialysis	
Number of patients	1403		284	
Age stratification	No (%)	Utility value Mean (SD)	No (%)	Utility value Mean (SD)
18-35 years	87 (6.20)	0.91 (0.13)	59 (20.77)	0.94 (0.10)
35-40 years	64 (4.56)	0.90 (0.14)	24 (8.45)	0.95 (0.09)
40-45 years	125 (8.91)	0.88 (0.16)	36 (12.68)	0.91 (0.13)
45-50 years	149 (10.62)	0.89 (0.15)	52 (18.31)	0.92 (0.12)
50-55 years	162 (11.55)	0.86 (0.18)	48 (16.90)	0.89 (0.17)
55-60 years	200 (14.26)	0.83 (0.19)	22 (7.75)	0.91 (0.22)
60-65 years	200 (14.26)	0.77 (0.22)	20 (7.04)	0.86 (0.17)
65-70 years	158 (11.26)	0.77 (0.24)	10 (3.52)	0.81 (0.23)
≥ 70 years	258 (18.39)	0.67 (0.24)	13 (4.58)	0.61 (0.31)

Abbreviation: SD: standard deviation; No: number.

Supplementary Table 3. Comparison of demographic and clinical characteristics between dialysis patients after 1:1 matching for individual characteristics, employment status, major comorbidities, and propensity score stratified by hemodialysis (HD) and peritoneal dialysis (PD).

Characteristics	HD	PD	di
**Number of patients**	144	144	
**Dialysis duration (month)**	48.7 (37.1)	40.4 (34.2)	23.26
**Age, Mean (SD)**	50.9 (12.3)	50.2 (12.5)	5.64
**No (%)** 18-34 years	15 (10.4)	14 (9.7)	2.33
35-49 years	62 (43.1)	59 (41.0)	4.26
50-64 years	47 (32.6)	54 (37.5)	10.28
65-79 years	18 (12.5)	14 (9.7)	8.92
≥ 80 years	2 (1.4)	3 (2.1)	5.34
**Sex (male, %)**	71 (49.3)	71 (49.3)	0.00
**Level of education, No (%)**			
Did not finish school	15 (10.4)	4 (2.8)	30.98
Elementary school	39 (27.1)	29 (20.1)	16.54
Junior high school	25 (17.4)	28 (19.4)	5.16
Senior high school	47 (32.6)	45 (31.3)	2.79
University and above	18 (12.5)	38 (26.4)	35.67
**No. of married (%)**	105 (72.9)	103 (71.5)	3.13
**Employed, No (%)**	79 (54.9)	79 (54.9)	0.00
**Comorbidities, No (%)**			
Diabetes mellitus	46 (31.9)	46 (31.9)	0.00
Hypertension	121 (84.0)	123 (85.4)	3.89
Cardiovascular disease	19 (13.2)	19 (13.2)	0.00
Stroke	3 (2.1)	3 (2.1)	0.00
Hyperlipidemia	22 (15.3)	22 (15.3)	0.00
Rheumatological disease	3 (2.1)	1 (0.7)	11.94
Chronic liver disease	17 (11.8)	17 (11.8)	0.00

Abbreviations: SD: standard deviation; di: standardized difference.

[*] Clinical characteristics used for matching are including age (± 5 years), sex, duration of dialysis (± 3 months), **employment status,** diabetes mellitus, cardiovascular disease, stroke, chronic liver disease, and propensity score for PD (± 0.05).

Supplementary Table 4. Comparison of frequency distributions of EQ-5D domain scores, utility values and visual analogue scales (VAS) between dialysis patients after 1:1 matching for individual characteristics, employment status, major comorbidities, and propensity score stratified by hemodialysis and peritoneal dialysis.

	**Hemodialysis**	**Peritoneal dialysis**	**di**
**EQ-5D score**	**N=144**	**N= 144**	
**Mobility dimension, %**			
No problems	111 (77.1)	120 (83.3)	15.61
Some problems	30 (20.8)	22 (15.3)	14.34
Confined to bed	3 (2.1)	2 (1.4)	5.34
**Self-care dimension, %**			
No problems	122 (84.7)	126 (87.5)	8.10
Some problems	15 (10.4)	12 (8.3)	7.22
Unable to wash/dress	7 (4.9)	6 (4.2)	3.36
**Usual activities dimension, %**			
No problems	104 (72.2)	117 (81.3)	21.67
Some problems	32 (22.2)	21 (14.6)	19.71
Unable to perform	8 (5.6)	6 (4.2)	6.49
**Pain/discomfort dimension, %**			
None	101 (70.1)	93 (64.6)	11.75
Moderate	42 (29.2)	47 (32.6)	7.36
Extreme	1 (0.7)	4 (2.8)	16.07
**Anxiety/depression dimension, %**			
None	111 (77.1)	106 (73.6)	8.13
Moderate	33 (22.9)	36 (25.0)	4.92
Extreme	0 (0.0)	2 (1.4)	16.85
**Utility score by TW function**			
Mean (SD)	0.88 (0.17)	0.88 (0.18)	0.00
Median	0.92	0.92	
Range	0.18-1.00	0.18-1.00	
**Utility score by UK function**			
Mean (SD)	0.89 (0.14)	0.89 (0.16)	0.00
Median	0.91	0.90	
Range	0.27-1.00	0.15-1.00	
**EQ-5D VAS, mean (SD)**	67.8 (13.1)	69.9 (14.6)	15.14

Abbreviations: SD: standard deviation; di: standardized difference; TW: Taiwan; UK: United Kingdom.

[*] Clinical characteristics used for matching were including age (± 5 years), sex, duration of dialysis (± 3 months), **employment status**, diabetes mellitus, cardiovascular disease, stroke, chronic liver disease,

and propensity score for peritoneal dialysis (± 0.05).

Supplementary Table 5. Comparison of demographic and clinical characteristics between 2:1 matching of hemodialysis (HD) and peritoneal dialysis (PD) patients for individual characteristics, major comorbidities, and propensity score*.

Characteristics	HD	PD	di
**Number of patients**	6984	3492	
**Dialysis duration (month)**	47.3 (30.2)	47.6 (30.7)	0.99
**Age, No (%)**			
**Mean (SD)**	56.1 (13.0)	56.0 (13.1)	0.77
18-34 years	361 (5.2)	176 (5.0)	0.91
35-49 years	1793 (25.7)	919 (26.3)	1.37
50-64 years	2937 (42.0)	1465 (42.0)	0.00
65-79 years	1611 (23.1)	793 (22.7)	0.95
≥ 80 years	282 (4.0)	139 (4.0)	0.00
**Sex (male, %)**	3300 (47.3)	1650 (47.3)	0.00
**Urbanization status**			
0 (Rural)	1794 (25.7)	897 (25.7)	0.00
1 (Satellite)	1972 (28.2)	986 (28.2)	0.00
2 (metropolitan)	3218 (46.1)	1609 (46.1)	0.00
**Comorbidities, No (%)**			
Diabetes mellitus	3330 (47.7)	1665 (47.7)	0.00
Hypertension	6099 (87.3)	3154 (90.3)	9.52
Cardiovascular disease	3453 (49.4)	1666 (47.7)	3.40
Congestive heart failure	1530 (21.9)	765 (21.9)	0.00
Coronary artery disease	2139 (30.6)	1094 (31.3)	1.51
myocardial infarction	150 (2.2)	75 (2.2)	0.00
Cardiac dysrhythmias	966 (13.8)	469 (13.4)	1.17
Peripheral vascular disease	895 (12.8)	349 (10.0)	8.82
Stroke	788 (11.3)	394 (11.3)	0.00
Hyperlipidemia	3358 (48.1)	1933 (55.4)	14.65
Rheumatological disease	442 (6.3)	213 (6.1)	0.83
Chronic liver disease	1526 (21.9)	763 (21.9)	0.00

Abbreviation: SD: standard deviation; di: standardized difference.

* Clinical characteristics used for matching were including age (± 2 years), sex, index year of initiation of dialysis, urbanization status, and major co-morbidities, including diabetes mellitus, acute myocardial infarction, congestive heart failure, stroke, chronic liver disease, and the propensity scores (± 0.05) for PD, which included hypertension, coronary artery disease, cardiac dysrhythmias, peripheral vascular disease, hyperlipidemia, and rheumatological disease.

Supplementary Table 6. Comparison of cost-effectiveness for maintenance hemodialysis (HD) and peritoneal dialysis (PD): Lifetime survival functions were estimated from 2:1 matched national cohorts (numbers of HD and PD: 6984 and 3492, respectively) based on 14 years of follow-up plus extrapolation, and utility values measured for current patients after matching (n=179 pairs).

	HD	PD	p-value
Life expectancy (years)	19.03 (0.09)	18.01 (0.10)	<0.001
Lifetime cost of NHI ± SE (US dollars)	245,663±4,633*	176,864±8,075*	<0.001
QALE ± SE (in QALY)	16.33 (0.4)	16.43 (0.28)	0.839
Cost per QALY ± SE (US dollars)	17,387±473*	12,359±600*	<0.001
ICER (PD-HD)	-79,986.4* (PD dominance)		

Abbreviation: NHI: National Health Insurance; SE: standard error; US: United States; QALE: quality-adjusted life expectancy; QALY: quality-adjusted life year; ICER: incremental cost-effectiveness ratio.

* The currency exchange rate is based on the value reported by the Central Bank of Taiwan on 2010/12/31: 1 United States Dollar = 30.368 New Taiwan Dollar. The gross domestic product per capita of Taiwan in 2010 is 18,573 US Dollar.

Supplementary Table 7. Comparison of EQ-5D mean utility values between the total cross-sectional samples of dialysis patients and the general population either before or after matched by age and sex.

	**Total cross-sectional samples** (n=1687)	**General population** (n=6052)	P value
**Utility values by TW function**			
Mean (SD)	0.824 (0.210)	0.936 (0.129)	< 0.001
	**Total cross-sectional samples** (n=1687)	**Age- and sex-matched general population** (n=1687)	
**Utility values by TW function**			
Mean (SD)	0.824 (0.210)	0.931 (0.131)	< 0.001

Abbreviation: SD: standard deviation; TW: Taiwan.

Supplementary Figure 1. The comparison of survival curves between 2:1 matched hemodialysis (HD) and peritoneal dialysis (PD) patients during a 14-year follow-up period.

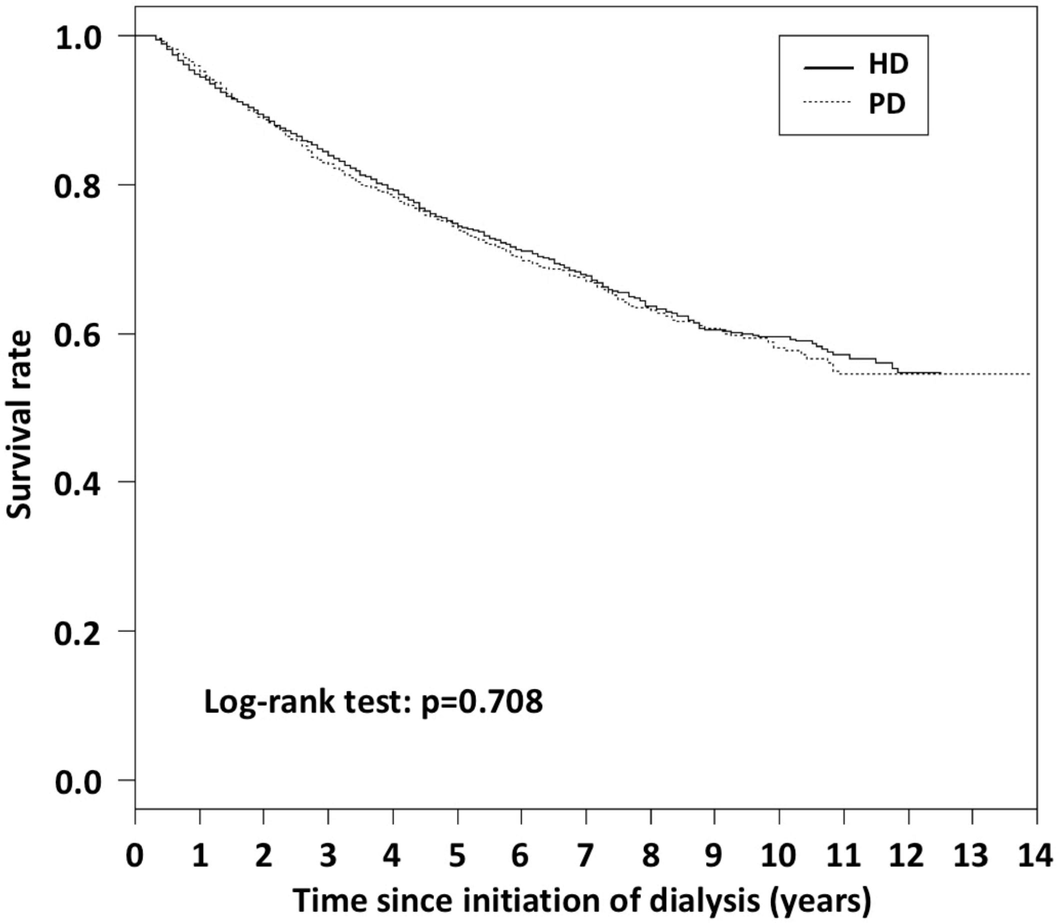

Supplementary Figure 2. (a) Average monthly costs for inpatient and outpatient healthcare expenditures and (b) monthly proportions of outpatient costs within total healthcare expenditures of patients selected from 2:1 matched hemodialysis (HD) and peritoneal dialysis (PD) during the follow-up period.

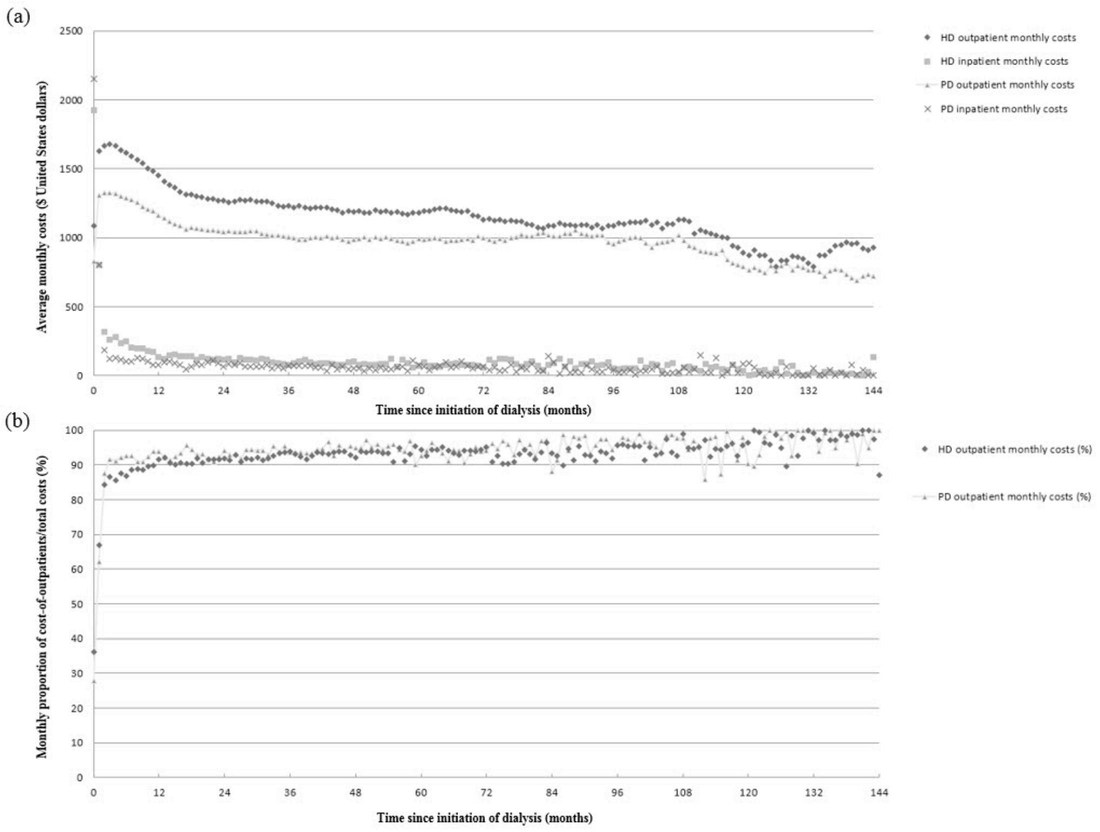

Supplementary Figure 3. The comparison of survival curves between 1:1 matched hemodialysis (HD) and peritoneal dialysis (PD) patients during a 14-year follow-up period.

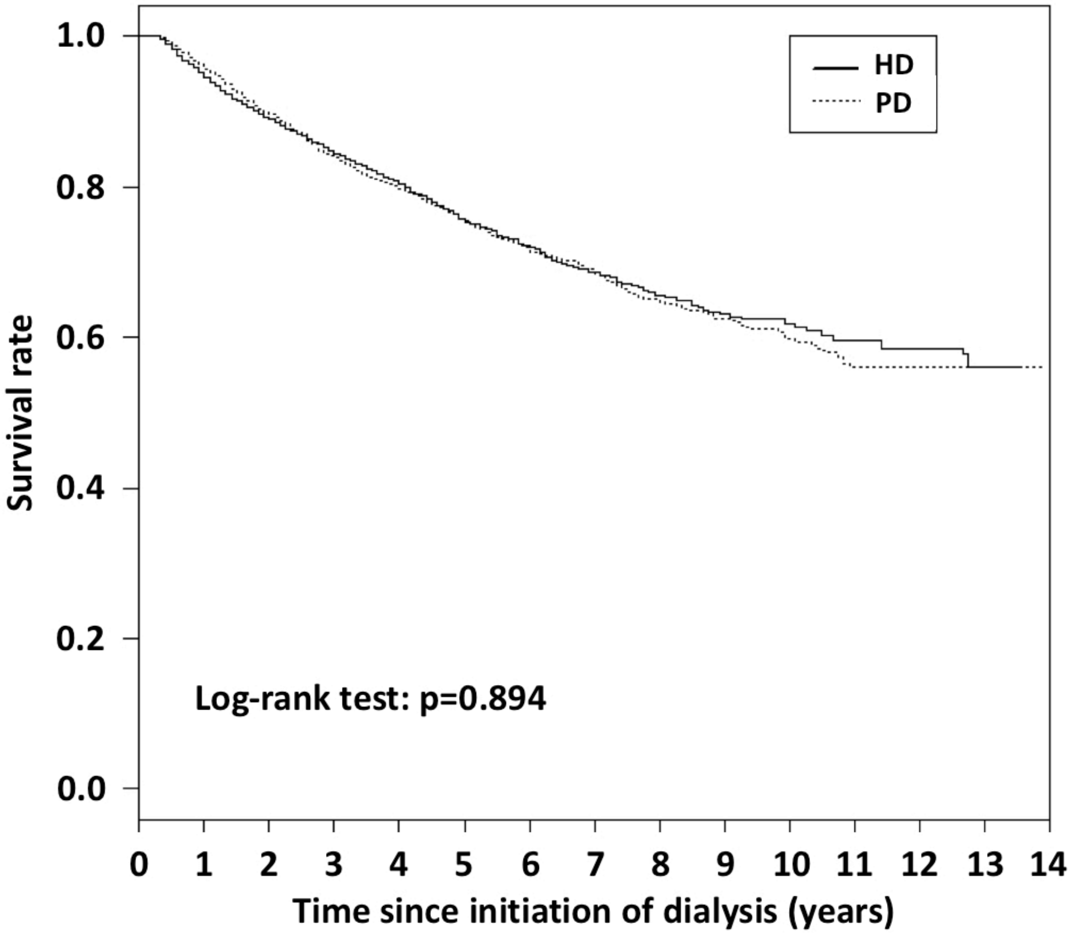

Supplementary Figure 4. Dynamic changes of mean EQ-5D utility values after initiation of dialysis estimated by kernel smoothing method for 179 pairs of matched patients under hemodialysis (HD, left panel) and peritoneal dialysis (PD, right panel) who were collected cross-sectionally from 12 dialysis units.

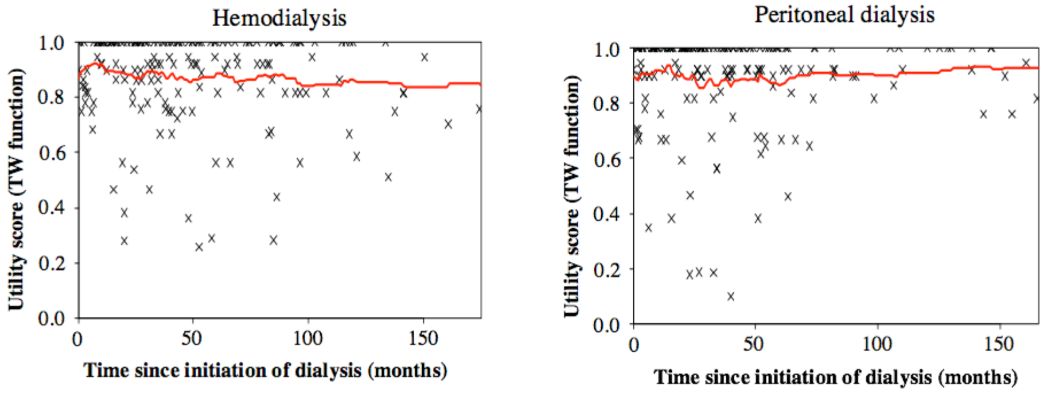

Supplement: Supplementary Information [file srep30266-s1.pdf]
